# Advancing imprinted polymers: Pre-orientation, self-assembly, and porosity in 3D printing

**DOI:** 10.1016/j.isci.2025.114378

**Published:** 2025-12-11

**Authors:** Amelie Huber, Benedikt Keitel, Sherman Lesly Zambou Jiokeng, Boris Mizaikoff, Mehmet Dinc

**Affiliations:** 1 Hahn-Schickard, Sedanstraße 14, 89077 Ulm, Germany; 2 Institute of Analytical and Bioanalytical Chemistry, Ulm University, Albert-Einstein-Allee 11, 89081 Ulm, Germany

**Keywords:** Chemistry, Molecular imprinted technique, Materials science, Polymers

## Abstract

Targeted isolation of specific compounds from complex matrices remains a central challenge across analytical, biomedical, and environmental sciences. Molecularly imprinted polymers (MIPs) offer a promising solution by enabling specific and reproducible binding of analytes. However, conventional imprinting techniques often suffer from heterogeneous binding sites and limited spatial control, restricting their broader utility. We see great potential in combining polymerization-induced phase-separation with photocuring 3D printing and oriented imprinting to fabricate monolithic, hierarchically porous MIPs with homogeneous and accessible binding moieties. This emerging platform enables the design of highly tailorable materials featuring distinct specificity and tunable architectures while being straightforward to produce, adapt, and scale. Beyond addressing current limitations, this strategy opens new avenues for translating imprinting technologies into industrially relevant formats. In this perspective, we outline the conceptual and technological implications of 3D-printed oriented MIPs, discuss their potential across disciplines, and highlight key challenges and opportunities for future research and application.

## Graphical abstract

**Figure:**
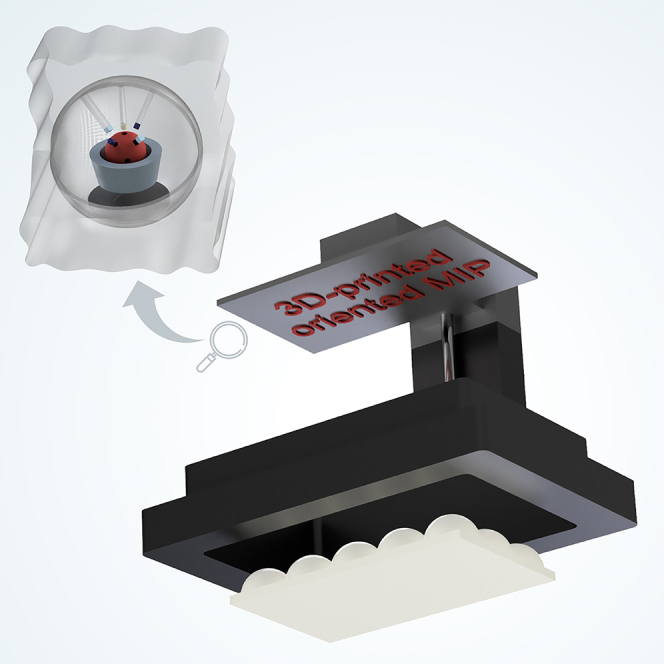

## Introduction

In the search for a synthetic approach to mimic the specific lock-and-key principle of receptors in nature, molecularly imprinted polymers (MIPs), also known as artificial receptors, were developed.^1^^,^^2^^,^^3^^,^^4^^,^^5^^,^^6^ MIPs are highly cross-linked polymeric matrices containing tailor-made binding sites complementary to the shape, size, and distribution of functional groups of the target analyte, referred to as the template.^7^ For the fabrication of such biomimetic materials, a substance of interest is used as a structure-directing template during the copolymerization of suitable monomeric building blocks, i.e., functional monomers and cross-linkers, in a well-adjusted porogen system. After extraction of the template, the complementary binding sites remain in the polymer to yield a receptor-like structure for the template.^8^

Compared to their biological analogs, e.g., receptors or antibodies, MIPs show similar specificity toward analytes^9^ while offering higher stability in varying pH values and temperatures,^7^ being facile and usually inexpensive in preparation,^10^ and allowing the use of several analytes and reagents during synthesis and analyses.^11^^,^^12^ These characteristics predestine MIPs for different applications, for example, as highly specific separation substances,^9^ as recognition elements in sensors,^13^ in drug-delivery systems,^14^ and in catalysis.^15^

Molecular imprinting techniques have evolved to include not only traditional methods such as bulk polymerization and sol-gel processes but also more recent approaches like suspension, emulsion, seed, and precipitation polymerization.^7^^,^^16^^,^^17^^,^^18^ MIPs can feature recognition sites within the bulk matrix, in a core-shell architecture, or confined to the surface.^1^^,^^3^^,^^19^^,^^20^^,^^21^^,^^22^^,^^23^ Bulk imprinting, the classical approach, involves polymerization into monolithic structures that require grinding prior to use, with binding sites randomly distributed throughout the matrix. While this method is simple and compatible with a wide range of monomers and templates, it often suffers from slow mass transfer rates and significant template leaching, which limits specificity and overall binding capacity.^3^^,^^24^ In contrast, core-shell and surface-imprinted MIPs feature binding sites localized at or in close proximity to the surface of a supporting substrate, allowing for faster binding kinetics due to reduced diffusion distances, minimized template leaching, and enhanced binding capacities.^1^^,^^10^^,^^21^^,^^22^^,^^23^

**Figure 1:**
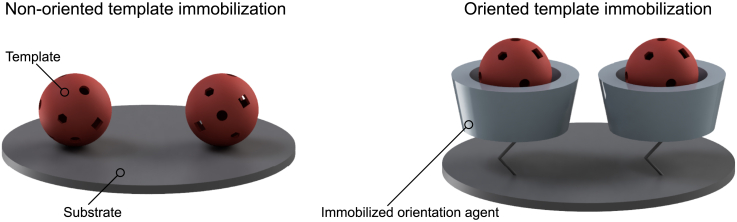
Non-oriented template immobilization in contrast to oriented template immobilization using an orientation agent

Nevertheless, these imprinting techniques often result in uncontrolled binding site formation, as the functional monomer-template complexes are fixed randomly arranged within the polymer matrix or within the polymer-shell.^25^^,^^26^ To achieve homogeneously oriented self-assembly between functional monomers and the template prior to imprinting, oriented template immobilization onto a surface is the strategy of choice (Figure 1).^27^^,^^28^

**Figure 2:**
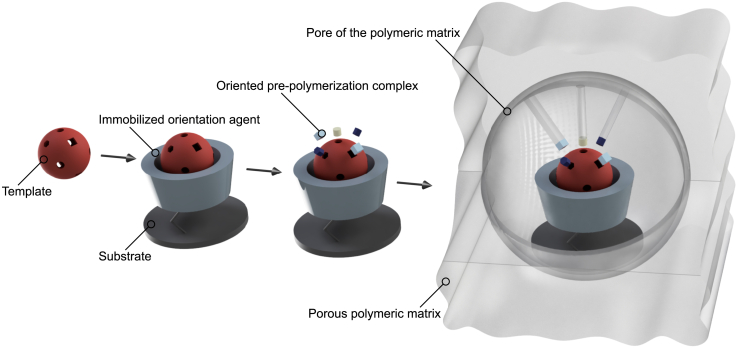
Schematic 3D representation of the production process of oriented MIPs

The utilization of an orientation agent that forces the template in a specific spatial orientation prior to imprinting is referred to as oriented imprinting. After anchoring the template, the remaining functional groups are reproducibly accessible to functional monomers, enabling spatially controlled covalent or non-covalent interactions. This results in the formation of homogeneous binding sites upon polymerization and ultimately improves the imprinting efficiency (Figure 2), as they offer superior binding specificity.^27^^,^^29^

In literature, different oriented imprinting strategies using various orientation agents for template fixation are reported, such as inhibitors,^30^ glutaraldehyde,^31^^,^^32^ boronates,^25^^,^^27^^,^^33^^,^^34^^,^^35^^,^^36^^,^^37^^,^^38^ aptamers,^27^ and chelating agents.^39^^,^^40^ The orientation agents can be immobilized on several types of substrates, such as (nano)particles,^28^^,^^31^^,^^32^^,^^35^^,^^38^^,^^39^^,^^40^^,^^41^^,^^42^^,^^43^ 96-well microplates,^33^ silicon wafers,^36^ and monolithic capillaries.^36^ Particle-based substrates could be based, e.g., on quantum dot (QD),^35^ metal-organic framework (MOF),^42^ silica (SiO_2_),^32^^,^^38^^,^^39^^,^^43^ or magnetic (Fe_3_O_4_)^28^^,^^31^^,^^38^^,^^40^^,^^41^ particles. Like their conventional counterparts, oriented MIPs have applications in various fields such as separation techniques,^44^ sensing,^45^ drug-delivery systems,^46^ and catalysis.^47^

Parallel to advances in molecular imprinting, additive manufacturing (AM), especially 3D polymer printing, gained increasing importance, driven by its exciting applications and rapid technological progress. As a key player in the manufacturing revolution, AM addresses major trends like customization and small-batch production.^48^ This method allows the manufacturing of complex structures in an additive point-by-point, line-by-line, or layer-by-layer polymerization process, whereby the shape and dimensions of the object to be printed are taken from a 3D computer-aided design (CAD).^49^^,^^50^^,^^51^^,^^52^ Today, AM includes many different technologies, such as photopolymerization (also known as photocuring or photo-cross-linking) techniques like digital light processing (DLP), stereolithography (SLA), continuous liquid interface production (CLIP), and liquid crystal display (LCD) masked photocuring technology.^50^^,^^53^^,^^54^ These technologies allow fine-tuning of the physicochemical and mechanical properties of the final polymer object by selecting appropriate precursors and processing conditions. Among the notable advantages of 3D printing are its design flexibility, reduced production times, demand-driven manufacturing, and enhanced industrial sustainability through optimized processes.^48^ Due to its versatile and tunable properties, 3D printing is nowadays widely used, e.g., in microfluidics,^50^ biomedicine,^50^ soft robotics,^55^ surgery,^56^ (tissue) engineering,^57^ dentistry,^57^ and cell cultivation.^58^ The technology of 3D printing is increasingly finding its place in the field of molecular imprinting, enabling the direct fabrication of application-tailored functional materials.^12^^,^^59^^,^^60^^,^^61^ Among photocuring methods, LCD-based 3D printing offers distinct advantages over point-based techniques, which, despite their high resolution, suffer from slow printing speeds and high costs. LCD-masked photocuring enables fast, layer-wide polymerization with sufficient resolution and structural fidelity. Additionally, LCD-based 3D printers are cost-effective and often easy to adapt for experimental use.^59^

Recently, we fabricated 3D-MIPs by polymerization-induced phase-separation (PIPS) in a scalable and reproducible LCD-masked photocuring AM process.^59^ In this study, monolithic, hierarchically porous substrates with complex macroscopic 3D designs and with excellent binding specificity toward the used template (i.e., 17*β*-estradiol), were generated. The term “hierarchically porous” refers to a combination of macroscopic pores obtained by using a digital printing file and microscopic pores resulting from the use of PIPS-promoting porogens.

Broadening the use cases of oriented MIPs, we see an unrealized potential in combining this concept with PIPS in photocuring AM technology, especially LCD-masked photocuring technology.

## Perspective

**Figure 3:**
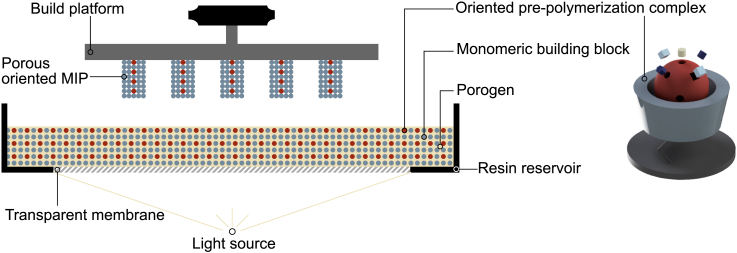
Manufacturing process of porous 3D-printed MIPs with oriented binding sites

Oriented 3D-printed MIPs could be fabricated by implementation of orientation agent-template composites during the polymerization of PIPS promoting resins in LCD-masked photocuring processes, leading to highly specific materials with tunable macroscopic (added 3D features in the 3D model) and inherent porosity, allowing enhanced mass transfer and spatial recognition of small and large molecules such as proteins (Figures 3 and 5).

**Figure 4:**
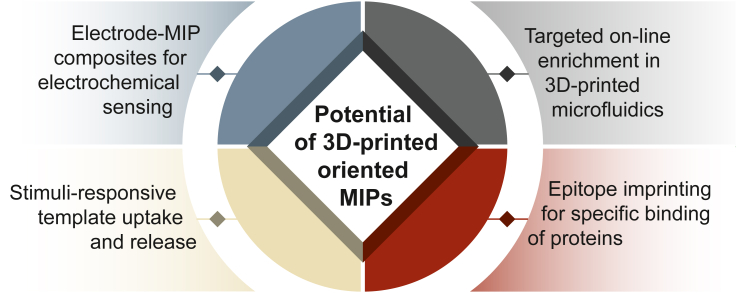
Possible fields of application for 3D-printed oriented MIPs

Substrate immobilized orientation agent-template complexes can be either homogeneously dispersed in the photocurable resin containing suitable monomers and PIPS-promoting porogen(s) (as depicted in Figure 3), or directly mounted on the surface of the build platform. In both cases, self-assembly with the functional monomers occurs, forming a spatially organized pre-polymerization complex between the template and the functional monomers. Upon photocuring, the solid substrates are expected to migrate to the interfaces of the two liquid phases (polymer-rich and polymer-poor phase) according to the pickering effect, resulting in 3D-designed porous MIPs that carry the substrates at the boundaries where the pores intersect the surrounding polymer.^62^ For rebinding of the template, the composite material must be removed, exposing the 3D-fixed binding sites adjacent to the resulting cavity complementary to the shape and size of the substrate. The 3D-printed MIP consequently could show porosity on three different levels, as it possesses macroscopic “printed” porosity (resulting from its applied CAD), a pore-network emerging from PIPS, and additional cavities with active binding sites due to removal of the orientation agent-template composite. All three types of porosity could be tailored either by adjusting the CAD, the amount and type of porogen(s), or the substrate, leading to an enhanced mass transfer and rendering this synthesis strategy even more interesting for various applications (Figure 4).

**Figure 5:**
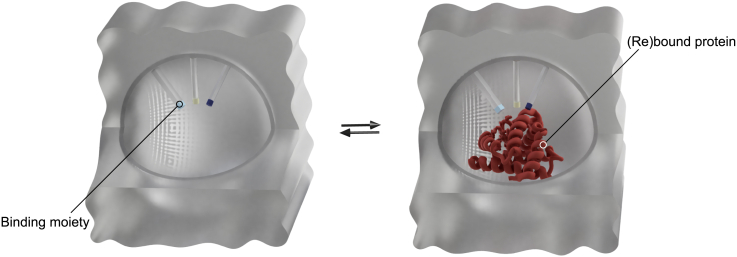
Binding process of a protein to the corresponding binding sites within the 3D-printed hierarchically porous oriented MIP

By employing this approach, direct printing of MIPs onto electrodes may become feasible, thus streamlining the process of electrochemical sensing.^63^^,^^64^^,^^65^^,^^66^^,^^67^ Moreover, MIPs could serve as specific target elements within microfluidic systems, enabling on-line analyte enrichment. In this scenario, the entire microfluidic setup could be printed as ready to use.^68^^,^^69^^,^^70^ Since the feasibility of multi-material vat photopolymerization has been established, this concept could be extended to the fabrication of MIP-assisted fully 3D-printed integrated sensors.^54^ Furthermore, by performing 4D printing, thus integrating stimuli-responsiveness, it could be possible to create polymers that respond to external stimuli, such as pH, temperature, or the addition of a solvent. This could lead to physicochemical alterations in the scaffold, potentially resulting in the release or binding of a template molecule.^71^^,^^72^^,^^73^ Herein, we especially want to highlight the potential for epitope imprinting, as this is, up to now, a challenging field.^74^ With the presented method, we expect that monolithic, hierarchically porous, oriented epitope-imprinted polymers with active binding sites that favor specific orientation for peptide binding, can be generated. These polymers can presumably rebind the corresponding, comparatively more spatially demanding biomacromolecules since the binding pockets are accessible not only through the pore network but also via the remaining voids derived upon removal of the substrate for pre-orientation (Figure 5).^48^^,^^75^^,^^76^^,^^77^

A real-world example of the potential of monolithic, hierarchically porous 3D-printed oriented MIPs in the biomedical field could be the transfer to additively manufactured nasopharyngeal swabs (NPSs). During the COVID-19 pandemic local and global supply chain disruption of essential biomedical devices, such as NPSs for SARS-CoV-2 sampling, which is the reference sampling method recommended by the WHO, occurred.^78^ Since 3D printing is already established in biomedical fields, the fabrication of additively manufactured biocompatible NPSs was suggested to meet the supply chain needs in addition to the mass production of conventional flock-head swabs. This technology enables rapid prototyping and cost-effective production on demand, while ensuring biocompatibility, safety, accuracy, comfort, thermal resistance, (design) flexibility, ease of handling, and functionality. Moreover, open-source collaborative design, enabled by the global exchange of digital models between academia and industry, accelerated the iterative innovation and optimization of both production and performance of 3D-printed NPSs. Studies have shown that these NPSs outperform conventional NPSs in terms of mucus collection and retention, as well as mechanical properties for greater comfort during sampling.^78^^,^^79^^,^^80^ We see great potential in introducing the proposed 3D printing process of oriented MIPs for virus binding into the AM of NPSs. This facilitates further functional improvements of these innovative biomedical devices by enabling affordable and accessible production of specific and selective swabs.

We anticipate that this method will have a tremendous impact on the current production of biomimetic receptors, as it could enable their simple, flexible, demand-driven, reproducible, and scalable production. The templates to be imprinted could correspond to ions, (small) molecules like, e.g., active pharmaceutical ingredients, or even epitopes for binding of larger entities, such as proteins, viruses, and bacteria, while the monomeric building blocks of the photocurable resin could be based on, e.g., acrylates or epoxides. The outstanding innovation of this approach is not only that it renders oriented imprinting easy to realize during on demand 3D printing, but in particular that it enables the production of 3D monolithic biomimetic receptors for large molecules, such as proteins or even viruses.

## Future perspectives

We are confident that by combining the concepts of photocuring AM, PIPS, and oriented imprinting, hierarchically porous 3D structures with remarkable binding affinity and analyte specificity can be prepared in a straightforward, tailored, scalable, demand-driven, and reproducible manner, rendering them highly versatile for a broad range of uses. This proposed strategy demonstrates the powerful combination of mechanical performance, determined by the structure and morphology generated by AM, and the functionality, introduced by oriented MIPs in terms of analyte specificity. The innovative MIP preparation method allows new possibilities for the development of isolation processes (in both gas and liquid phase), sensors, and catalysts. Looking ahead, the integration of this methodology with emerging technologies, such as 4D printing, microfluidics, and epitope imprinting holds promise for even broader applications in industry. Utilizing the versatility of 4D printing could enable dynamic responses to external stimuli, enhancing the functionality of MIP-based devices. Microfluidics integration may facilitate on-line specific enrichment and separation of analytes from complex matrices in a single step production method generating so-called MIP-on-a-chip devices. Furthermore, oriented epitope imprinting could be used for the specific binding of biomacromolecules or even viruses or cells on a large scale. In essence, the presented combination of these methodologies could provide groundbreaking advances in diverse fields ranging from healthcare and environmental monitoring to industrial catalysis and beyond.

Future studies should comprehensively evaluate the overall performance of 3D-printed oriented MIPs. This includes assessing and optimizing the compatibility between orientation agents and substrates across diverse templates, as well as verifying binding site homogeneity using surface or spectroscopic analyses. The mass transfer within the hierarchically porous structures should be modeled to enable rational structural design, while mechanical tests must be performed to determine the mechanical stability and robustness of the functional materials. In addition, the reproducibility of the developed hierarchically porous oriented bulk MIPs throughout different batches and printing conditions should be investigated. The validation of the MIP performance should also be performed in complex real-world matrices, including for instance serum, urine, or wastewater and must be benchmarked against conventional MIP counterparts.

Application-oriented investigations should further focus on evaluating the rebinding properties and release mechanisms of target analytes under real-time sensing conditions. In biomedical applications, such as the AM of NPSs, the biocompatibility of the oriented epitope-imprinted polymers must be confirmed, analyzed, and optimized.

## Acknowledgments

This work was financially supported by the EU Horizon 2020 project ENVIROMED (grant no. 101057844), which focuses on developing a next-generation toolbox for greener pharmaceutical design and manufacturing to reduce environmental impact. Furthermore, it was partially funded by ZIM (Zentrales Innovations programm Mittelstand) under the project MIPextract (grant KK5054610SK1).

## Author contributions

Conceptualization, all authors; writing—original draft, A.H.; writing—review & editing, all authors except A.H.; visualization, A.H.; funding acquisition, B.M.; resources, B.M. and M.D.; project administration, B.M. and M.D.; supervision, B.M. and M.D.

## Declaration of interests

B.K., B.M., and M.D. are co-inventors on a patent application related to the LCD-based 3D-printed MIP technology described in this manuscript (PCT/EP2024/076002). One of the authors of this study, B.M., is a guest editor of the special issue “Translational and interdisciplinary advances of molecular imprinting technology” to which this paper belongs.
